# Comparative vector competence of North American Lyme disease vectors

**DOI:** 10.1186/s13071-020-3893-x

**Published:** 2020-01-14

**Authors:** Lisa I. Couper, Youyun Yang, Xiaofeng Frank Yang, Andrea Swei

**Affiliations:** 1 Department of Biology, Stanford University, Stanford, CA USA; 2 Department of Microbiology and Immunology, Indiana University School of Medicine, Indianapolis, IN USA; 3 Department of Biology, San Francisco State University, San Francisco, CA USA

**Keywords:** Lyme disease, *Ixodes scapularis*, *Ixodes pacificus*, Vector competence, Pathogen acquisition, Pathogen transmission

## Abstract

**Background:**

Understanding the drivers of Lyme disease incidence at broad spatial scales is critical for predicting and mitigating human disease risk. Previous studies have identified vector phenology and behavior, host community composition, and landscape features as drivers of variable Lyme disease risk. However, while the Lyme disease transmission cycles in the eastern and western USA involve different vector species (*Ixodes scapularis* and *Ixodes pacificus*, respectively), the role of vector-specific differences in transmission efficiency has not been directly examined. By comparing the performance of traits involved in vector competence between these two species, this study aims to identify how vector competence contributes to variable Lyme disease risk.

**Methods:**

We used a suite of laboratory experiments to compare the performance of traits related to vector competence for the two USA Lyme disease vectors. For each species, we measured the rate of attachment to a common rodent host, the engorgement weight, and the efficiency of pathogen acquisition (host to tick) and pathogen transmission (tick to host) from laboratory mice. In measuring pathogen acquisition and transmission, we used two different pathogen strains, one sympatric with *I. scapularis* and one sympatric with *I. pacificus*, to assess the importance of vector-pathogen coevolutionary history in transmission dynamics.

**Results:**

We found *I. pacificus* had significantly higher host attachment success and engorgement weights, but significantly lower pathogen transmission efficiency relative to *I. scapularis*. Molting success and pathogen acquisition did not differ between these two species. However, pathogen acquisition efficiency was significantly higher for both sympatric vector and pathogen strains than the allopatric pairings.

**Conclusions:**

This study identified species-specific vector traits as a potential driver of broad scale variation in Lyme disease risk in the USA. In particular, the exceedingly low rates of pathogen transmission from tick to host observed for *I. pacificus* may limit Lyme disease transmission efficiency in the western USA. Further, observed variation in pathogen acquisition between sympatric and allopatric vector-pathogen strains indicate that vector-pathogen coevolutionary history may play a key role in transmission dynamics. These findings underscore the need to consider vector traits and vector-pathogen coevolution as important factors governing regional Lyme disease risk.

## Background

Tick-borne diseases pose serious threats to public health in the USA as many diseases are increasing in incidence and geographical distribution, new pathogens are emerging, and new vector species are introduced [1, 2]. Despite efforts to manage tick populations and pathogen transmission, the most effective method for preventing human disease still relies on tick avoidance and prompt removal [3], necessitating a strong understanding of the spatial and temporal patterns of disease risk. Efforts to understand and predict spatial trends in tick-borne disease typically focus on regional disease systems as ticks are highly sensitive to local microhabitat conditions and host communities [4–8]. However, substantial spatial heterogeneity in human incidence exists at broad spatial scales. For example, reported cases of Lyme disease, the most common vector-borne disease in the USA, are scattered throughout the continental USA, but approximately 93% of cases occur in ten states in the Northeast and upper Midwest [9]. Despite the high spatial clustering of cases in these regions, the tick species responsible for vectoring Lyme disease are present in nearly half of all USA counties [10]. Identifying the factors underlying incidence across space, rather than vector presence alone, is thus critical for improved prediction and mitigation of human disease risk.

The divergent Lyme disease cycles in the eastern and western USA present an ideal system for studying ecological drivers of human incidence at broad spatial scales. While Lyme disease is endemic in both regions, the pathogen, *Borrelia burgdorferi* (*sensu stricto*), is maintained by different vector species in distinct enzootic cycles comprised of different reservoir hosts [11, 12]. In the eastern USA, *B. burgdorferi* transmission is maintained by a suite of small mammal hosts, most notably white-footed mice, least chipmunks, long-tailed and short-tailed shrews and bushy-tailed squirrels; and transmitted between hosts by the blacklegged tick, *Ixodes scapularis* [13]. In the western USA, *B. burgdorferi* is maintained primarily by the western gray squirrel, dusky-footed woodrat and deer mice, and vectored by the western blacklegged tick, *Ixodes pacificus* [14].

While both of these systems feature small mammal pathogen reservoirs and ixodid tick vectors, Lyme disease incidence in the eastern and western USA differs by orders of magnitude [15]. Prior reports suggest that ecological drivers of broad-scale differences in incidence include variation in tick host community composition [16, 17], vector phenology [18, 19] and differences in vector questing behavior [20, 21]. These factors may contribute to differences in incidence between the eastern and western USA, however, more fundamental differences in the competence of the two vector species involved, *I. scapularis* and *I. pacificus*, remain to be explored.

Prior comparisons of *I. scapularis* and *I. pacificus* indicate these species are genetically and morphologically similar [22, 23], have similar feeding and reproductive strategies [24], and similar host preferences in the laboratory [22]. Despite these similarities, these species exhibit key differences in their life histories, likely driven by the differing evolutionary and ecological conditions of their disjoint distributions. The distribution of *I. scapularis* extends across the eastern USA, with recent geographical expansions observed in the northeastern, north-central and midwestern USA, while the distribution of *I. pacificus* covers the far western USA and has remained relatively stable in recent decades [10]. Thus, the host communities and climatic conditions experienced by each species differ markedly, generating differences in their host usage [25–27], phenology [24] and climate sensitivities [28]. The impact of these life history differences on vector traits related to competence is largely unexplored but may help further explain broad scale geographical differences in Lyme disease incidence.

**Fig. 1:**
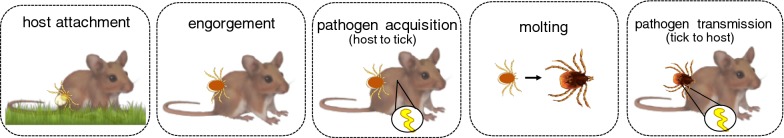
Vector traits involved in vector competence. The panels depict, in order, an unengorged larva (*I. scapularis* or *I. pacificus*) seeking a rodent host, an engorged larva on a rodent host, an engorged larva transmitting *B. burgdorferi* to a rodent host, an engorged larva molting to the nymphal life stage, and a nymph transmitting *B. burgdorferi* to a rodent host. Tick and mouse graphics illustrated by Mona Luo

Vector competence, the ability of a vector to acquire, maintain, and transmit pathogens underlies the efficiency of pathogen transmission [12, 29, 30]. Establishing vector competence for a particular tick species and pathogen requires successful execution of a sequence of steps [30] (Fig. 1, Table 1). Namely, a tick must find and attach to a competent host, acquire the pathogen during feeding, molt and maintain the pathogen during molting, then attach and transmit the pathogen to another host [31]. Given the number of steps involved, many vector-specific traits and extrinsic factors influence vector competence, enabling large variation in competence even in closely related species [32, 33]. However, comparisons of vector competence between species typically focus on one or two of the above steps [22, 34–36], obscuring the overall impact of vector competence on disease risk and hindering our ability to identify bottlenecks in transmission [37].

**Table 1:** Vector competence traits measured in *I. scapularis* and *I. pacificus*

Trait	Ecological significance	Result
Host attachment success	Successful attachment to a competent host, typically a small mammal, is necessary for potential pathogen acquisition [110]	*I. pacificus* had significantly higher host attachment success than *I. scapularis*
Feeding rate	Longer attachment to hosts can facilitate pathogen transmission, but may also stimulate host immune responses [111–113]	*I. scapularis* fed to repletion significantly faster than *I. pacificus*
Engorgement weight	Higher engorgement weights may reflect higher resource uptake and be associated with greater molting success and/or survivorship [76] but see [59]	*I. pacificus* had significantly higher engorgement weights than *I. scapularis*
Molting success	Successful larval molting is a prerequisite for pathogen transmission during the nymphal life stage [110]. Transstadial transmission is also required but has previously been measured at high rates for both *I. scapularis* [114, 115]	An equal proportion of *I. scapularis* and *I. pacificus* successfully molted
Pathogen acquisition (host to tick)	As *B. burgdorferi* is not vertically transmitted, pathogen acquisition by larvae is crucial for *B. burgdorferi* maintenance and amplification [12]	An equal proportion of *I. scapularis* and *I. pacificus* acquired their respective sympatric pathogen strain, and an equal but lower proportion of *I. scapularis* and *I. pacificus* acquired their allopatric pathogen strain
Pathogen transmission (tick to host)	As the adult vector life stages typically feed on non-competent reservoir hosts, efficient pathogen transmission by nymphs to hosts is critical for *B. burgdorferi* maintenance and amplification [12]	*I. scapularis* transmitted the pathogen to a greater number of hosts than *I. pacificus* which did not transmit to any hosts, although unequal numbers of infected ticks were applied to hosts

To investigate the role of vector competence in Lyme disease transmission at broad spatial scales, we compared the performance of the two USA vector species, *I. scapularis* and *I. pacificus*, in various ecological and physiological processes involved in pathogen dynamics. Using controlled laboratory manipulations, we measured the host attachment and feeding rate, engorgement weight, larval molting success, and efficiency of pathogen acquisition (tick to host) and pathogen transmission (host to tick) (Fig. 1).

## Methods

### Sample collection

Larval *I. scapularis* and *I. pacificus* used for vector competence comparisons were obtained from BEI Resources (BEI Resources, Manassas, VA, USA; NR-44115, NR-44387). Colonies of *I. scapularis* and *I. pacificus* originated from ticks collected from vegetation in Rhode Island in 2003, and California in 2000, respectively. Both species were then reared under identical, standard lab conditions, entailing maintenance in sterile polystyrene containers in environmental incubators at a relative humidity of 90%, temperature of 22 ± 1 °C, and a 16:8 h light:dark photoperiod [38, 39]. Deer mice, *Peromyscus maniculatus*, were used for all host feeding assessments as this species is a common tick host found in the ranges of both *I. scapularis* and *I. pacificus* [39, 40]. Relative feeding on *P. maniculatus* is generally lower for *I. pacificus* relative to *I. scapularis* [27, 41, 42] but in some regions such as the Midwest, the absolute mean burdens are similar [43, 44]. Further, *I. pacificus* will readily parasitize *P. maniculatus* when available [41–43] and the lower natural parasitism rates are likely due to a preference for lizards rather than physiological incompetence for mice-feeding [43]. Adult female *P. maniculatus bardii* were obtained from the Peromyscus Genetic Stock Center at the University of South Carolina and were identical in age, sex and prior husbandry. Statistical comparisons of vector competence measurements were assessed *via* two-proportion *Z*-tests or Kruskal–Wallis tests, conducted in R v3.4.3.

### Host attachment and feeding rate

The host attachment success of *I. scapularis* and *I. pacificus* was measured through laboratory feeding on *P. maniculatus.* Larval *I. scapularis* and *I. pacificus* were manually placed on naïve *P. maniculatus* that had been anesthetized with isoflurane. Two *P. maniculatus* were used for each tick species and 150 larvae were used per mouse for a total of 300 larvae of each tick species. After the initial placement of ticks on the mouse ears and neck, each mouse was held within a cloth bag for 16 h to provide increased opportunity for tick attachment [44]. After 16 h, mice were removed from the bag and maintained in a wire cage held above a water dish into which ticks dropped off and were collected daily. Replete ticks were transferred to sterile polystyrene containers as described above. During these procedures, room temperature was maintained at 22 ± 1 °C, and a 16:8 h light:dark photoperiod was used [39].

### Engorgement weight

The weights of successfully fed larval *I. scapularis* and *I. pacificus* were measured using a Mettler Toledo analytical balance (Mettler Toledo, Columbia, OH, USA) with precision to 0.1 mg. Due to low individual larval weights, larvae were weighed in batches of 5 and the batch weight was divided by 5 to obtain individual weights (*n* = 200, 40 *I. pacificus* batches; *n* = 230, 46 *I. scapularis* batches). Unengorged larval *I. scapularis* and *I. pacificus* from the same BEI stock as those used in the experiments were also measured to determine the weights for each species prior to feeding.

### Molting success

Successfully fed larval *I. scapularis* and *I. pacificus* were maintained in plastic vials at a humidity of 98% [42] and a 16:8 h light:dark photoperiod until individuals either molted to the nymphal life stage or died. Engorged larvae took approximately 2 months to molt during which time freshly molted ticks were moved to clean vials. Those that had not molted after 2 months and were immobile appeared to be covered in tick waste (tick feces, exuviae), which is common in the high humidity environment, and were presumed dead [45].

### Pathogen acquisition (host to tick)

Pathogen acquisition efficiency was measured in larval *I. pacificus* and *I. scapularis* through feeding ticks on infected C3H/HeN laboratory mice. To account for the potentially confounding effects of differences in regional *B. burgdorferi* strains, we conducted infection trials with mice infected with either CA4, a strain originally isolated from California [46], or B31, a strain from the Northeast [47] in a 2 × 2 factorial design. Both strains had been passaged 4–6 times in the laboratory before use. Mice were needle-inoculated with 10^5^ spirochetes/mouse [48] using live culture grown in BSK-II. To confirm mouse infection status, one 2 mm ear tissue biopsy was taken from each mouse, extracted for DNA using a Qiagen DNeasy extraction kit (Qiagen, Valencia, CA, USA), and tested for infection through a nested PCR targeting the 5S–23S intergenic spacer region of *Borrelia* [49]. Positive PCR results were typically obtained by 3–4 weeks post-inoculation. As described in the above section, *I. scapularis* and *I. pacificus* were fed on mice infected with CA4 or B31 and collected from drop-off containers. All ticks were then stored in 70% ethanol at − 80 °C and, within two weeks, individually extracted and tested for infection *via* the PCR described above.

### Pathogen transmission (tick to host)

Pathogen transmission efficiency from ticks to a blood-meal host was measured in nymphal *I. scapularis* and *I. pacificus* through feeding on naïve C3H/HeN mice in the laboratory. Transmission to more ecologically relevant host species, such as *Peromyscus* spp., could not be achieved due to exceedingly low attachment rates of *I. pacificus* nymphs to *Peromyscus* under laboratory conditions (Chindy Peavey, personal communication). Putatively infected ticks used for this experiment were obtained through larval feeding on the B31 or CA4-infected mice used in the pathogen acquisition experiment, and maintenance at 98% humidity in plastic vials in the laboratory until successful molting. Due to variable survival rates and numbers of successfully molted nymphs, we were unable to attach equal numbers of nymphs to mice (Additional file 1: Table S1). Nymphs were then fed to repletion on uninfected C3H/HeN mice. Mice were tested for infection as described above. Because it is impossible to know the infection status of ticks prior to host attachment, we confirmed tick infection status after host feeding by nested PCR. Only mice fed with at least 1 *post-hoc*, PCR-confirmed, infected tick were included in the final tick-to-host transmission analysis.

## Results

### Host attachment success and feeding time

**Fig. 2:**
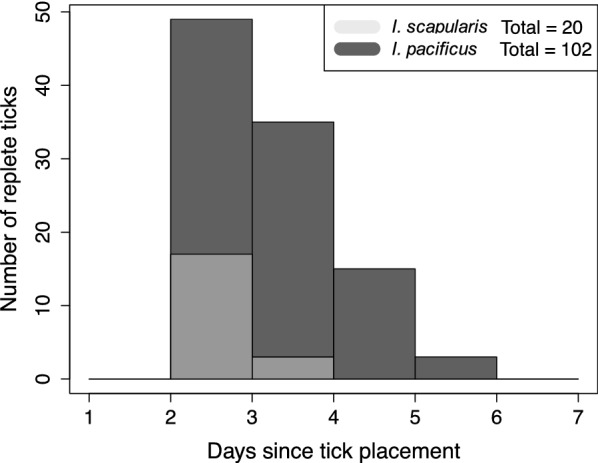
Host attachment and feeding rates of larval *I. scapularis* and *I. pacificus.* Bar heights indicate the number of ticks, out of 300 for each species, that fed to repletion on the subsequent 7 days after placement on *P. maniculatus*. Similarly, “Total” refers to the total number of larvae that successfully completed feeding

Of the 300 larvae of each tick species placed on *P. maniculatus*, 102 *I. pacificus* and 20 *I. scapularis* successfully attached and fed (Fig. 2). While *I. pacificus* had higher host attachment success, *I. scapularis* fed to repletion significantly faster (3.15 ± 0.37 days) than *I. pacificus* (3.73 ± 0.82 days) (*t*_(63)_ = 4.98, *P* < 0.01).

### Engorgement weight

**Fig. 3:**
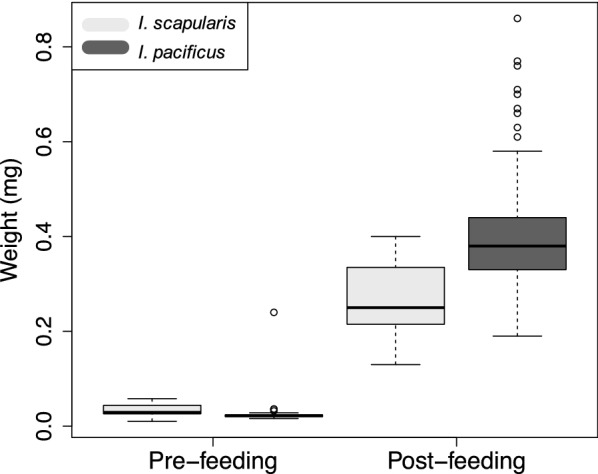
Larval *I. scapularis* and *I. pacificus* weights pre- and post-feeding to repletion on *P. maniculatus.* Horizontal bar and star denote statistically different weights (*P* < 0.01)

Engorged *I. pacificus* larvae weighed significantly more (0.412 ± 0.185 mg) than engorged *I. scapularis* larvae (0.265 ± 0.007 mg) (*t*_(29)_ = 5.47, *P* < 0.001) (Fig. 3, Additional file 2: Table S2) after feeding on *P. maniculatus*. Prior to feeding, there was no difference in the weights of larval *I. pacificus* (0.028 ± 0.001 mg) and *I. scapularis* (0.033 ± 0.0002 mg) (*t*_(47)_ = − 0.90, *P* = 0.37) (Fig. 3).

### Molting success

Of the engorged ticks maintained for possible molting, an equal proportion of *I. pacificus* (60/100, 60%) and *I. scapularis* (11/18, 61.1%) successfully molted to the next life stage (*χ*^2^ = 0.001, *df* = 1, *P* = 1) (Table 1). These counts were taken 2 months after drop-off when all ticks had either died or molted.

### Pathogen acquisition (host to tick)

**Fig. 4:**
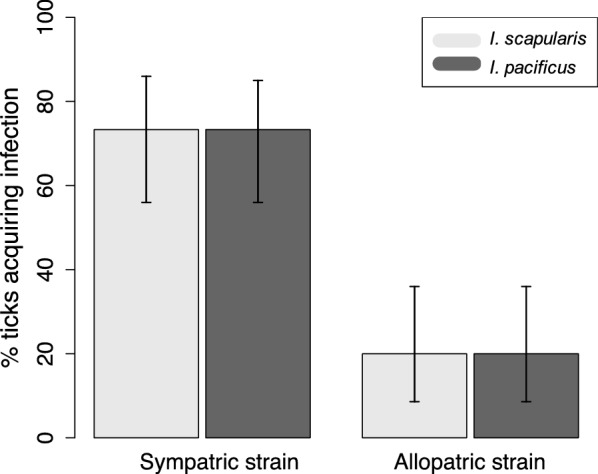
Percent of larval *I. scapularis* and *I. pacificus* acquiring *B. burgdorferi* from an infectious mouse blood meal. Mice were infected with *B. burgdorferi* strain B31 or CA4, the pathogen strains sympatric for *I. scapularis* or *I. pacificus*, respectively. Errors bars denote 95% credible intervals

We measured the infection status of 30 engorged larval ticks from each of the treatment groups (*I. pacificus* or *I. scapularis* fed on a B31 or CA4-infected mouse) to calculate pathogen acquisition percentages. We found 73% (22/30) of *I. scapularis* that fed on mice infected with B31, the northeastern pathogen strain, acquired infection, while only 20% (6/30) of *I. scapularis* acquired infection from mice infected with CA4, the western pathogen strain (Fig. 4). Similarly, 73% (22/30) of *I. pacificus* fed on CA4-infected mice acquired the pathogen, compared to 20% (6/30) of those fed on B31-infected mice (Fig. 4). Thus, pathogen acquisition was identical for each vector exposed to its sympatric pathogen strain, and identical but lower for allopatric pathogen strains.

### Pathogen transmission (tick to host)

We measured the infection status of mice 10 days after feeding by infected ticks from each treatment group to calculate pathogen transmission percentages. Pathogen transmission was higher from *I. scapularis* than *I. pacificus* for both pathogen strains (*χ*^2^ = 8.55, *df* = 1, *P* = 0.003) (Table 2). For *I. scapularis* nymphs infected with B31 or CA4, transmission to naïve mice occurred in 100% (5/5) or 83% (5/6) of mice, respectively. For *I. pacificus*, no mice became infected from either B31- or CA4-infected nymphs (0/2 mice and 0/3 mice, respectively). However, unequal numbers of infected *I. scapularis* and *I. pacificus* nymphs were applied to each mouse due to experimental difficulties and sample limitations of infected nymphs (see Table 2), which may confound conclusions about the transmission efficiency of individual ticks of each species.

**Table 2:** Pathogen transmission rates from *I. scapularis* and *I. pacificus* to naïve C3H/HeN mice

Mouse ID	Tick species	Pathogen strain	No. of feeding ticks^a^	Mouse infection status
**1**	***I. scapularis***	**B31**	**7/10**	**Infected**
**2**	***I. scapularis***	**B31**	**9/11**	**Infected**
**3**	***I. scapularis***	**B31**	**6/7**	**Infected**
**4**	***I. scapularis***	**B31**	**8/9**	**Infected**
**5**	***I. scapularis***	**B31**	**5/5**	**Infected**
**6**	***I. scapularis***	**CA4**	**3/6**	**Infected**
**7**	***I. scapularis***	**CA4**	**4/5**	**Infected**
**8**	***I. scapularis***	**CA4**	**2/6**	**Infected**
**9**	***I. scapularis***	**CA4**	**1/8**	**Infected**
**10**	***I. scapularis***	**CA4**	**5/5**	**Infected**
**11**	***I. scapularis***	**CA4**	**2/6**	**Not infected**
12	*I. pacificus*	B31	0/3	Not infected
13	*I. pacificus*	B31	0/4	Not infected
14	*I. pacificus*	B31	0/3	Not infected
15	*I. pacificus*	B31	0/3	Not infected
**16**	***I. pacificus***	**B31**	**1/1**	**Not infected**
**17**	***I. pacificus***	**B31**	**1/1**	**Not infected**
**18**	***I. pacificus***	**CA4**	**2/2**	**Not infected**
**19**	***I. pacificus***	**CA4**	**2/2**	**Not infected**
**20**	***I. pacificus***	**CA4**	**5/5**	**Not infected**

*Note*: Rows in bold indicate ticks fed on these mice were infected, while non-bold rows indicate ticks were uninfected

^a^Number infected/total number

## Discussion

Efficient vector-borne pathogen transmission cycles require competent vectors—arthropod species that can reliably acquire, maintain, and transmit pathogens [12, 29]. We decomposed vector competence into measurable vector traits: host attachment and feeding success; larval molting success; pathogen acquisition (host to tick); and pathogen transmission (tick to host). We compared performance of these traits between the two USA Lyme disease vectors, *I. scapularis* and *I. pacificus*, to investigate if variation in vector competence may contribute to broad geographical differences in Lyme disease incidence. Despite high genetic similarity between these two species, we detected significant differences in host feeding and pathogen transmission abilities, but no difference in larval molting success or pathogen acquisition rates (Table 1).

The choice and success of larval host feeding is a critical factor in Lyme disease transmission efficiency [22] as it presents the first opportunity for pathogen acquisition, and dictates the infection status of the nymphal stage, responsible for the majority of human Lyme disease cases [50, 51]. Natural host usage by *I. scapularis* and *I. pacificus* is known to be markedly different, with immature *I. scapularis* feeding more commonly on small mammals such as the white-footed mouse, *Peromyscus leucopus* [52, 53], and immature *I. pacificus* feeding predominantly on the western fence lizard, *Sceloporus occidentalis* [27, 43]. As small mammal hosts are generally more highly competent for *B. burgdorferi* [53–55], the rate of vectors attaching and successfully feeding on these hosts is an important factor in the Lyme disease transmission cycle [56]. In our experimental manipulation, we detected a five-fold greater abundance of *I. pacificus* attaching to and successfully feeding on a common small mammal host, *Peromyscus maniculatus*, compared to *I. scapularis* (Fig. 2). This finding supports previous observations that *I. pacificus* readily parasitize rodents when available [22, 43], despite more frequently parasitizing lizards in the field. These findings suggest that *B. burgdorferi* transmission in the western USA is not limited by *I. pacificus* host feeding abilities. Further, host community shifts favoring small mammals, or *I. pacificus* population expansions to areas with greater small mammal abundances relative to lizards, present opportunities for more efficient *B. burgdorferi* transmission cycles than currently observed. The relatively low attachment rates of *I. scapularis* on *P. maniculatus* observed here may not reflect *I. scapularis* attachment to all rodents, as *I. scapularis* more commonly parasitizes *P. leucopus* in the field [52, 53] rather than *P. maniculatus* used here. However prior laboratory experiments have demonstrated that *I. scapularis* attaches at least as readily to *P. maniculatus* as to *P. leucopus* [57].

In addition to higher host parasitism rates, we found *I. pacificus* larvae remained attached to hosts for longer (Fig. 2) and obtained higher engorgement weights than *I. scapularis* (Fig. 3). Although not directly measured here, attachment duration and tick engorgement can reflect host immune resistance to tick infestation [58–60]. Namely, tick feeding is known to induce a complex array of host immune responses [59, 61, 62]. These host immune responses can result in immunological resistance to tick infestation which can also confer host protection to tick-borne pathogens [63–66]. Development of this host immunological resistance has been associated with shorter host attachment times [64, 67, 68], decreased tick engorgement [58, 64, 67, 69, 70], and decreased tick parasitism rates [69, 71], but see [57, 72, 73]. Thus, the results obtained here may indicate enhanced host resistance to pathogens transmitted by *I. scapularis* than those from *I. pacificus*. Further, increased tick engorgement has been associated with greater tick molting success [74], fecundity [75], and larger molted body sizes or weights which may enhance survival [70, 74, 75]. However, engorged larval *I. scapularis* and *I. pacificus* molted at equally high rates in this study, and downstream effects on survival and fecundity were not measured here. Thus, the lower attachment duration and engorgement of *I. scapularis* relative to *I. pacificus* had no measured impact on vector survival, but could negatively impact tick fitness and pathogen transmission to hosts.

In contrast, results from the pathogen transmission experiment indicate that *B. burgdorferi* transmission was much higher from *I. scapularis* than *I. pacificus* (Table 2). Infected nymphal *I. pacificus* did not transmit either pathogen strain (B31 or CA4) to naïve laboratory mice, while *I. scapularis* transmitted B31 and CA4 to nearly all mice. As pathogen transmission from nymphal to vertebrate hosts is a key determinant of Lyme disease maintenance and human disease risk [17, 76, 77], the low transmission rates from *I. pacificus* represent a potential bottleneck in pathogen dynamics in the western USA. However, transmission to C3H/HeN laboratory mice used in this experiment may not reflect transmission dynamics to *Peromyscus* or other small mammal species naturally encountered by *I. scapularis* and *I. pacificus.* Specifically, pathogen reservoir hosts for *I. pacificus* transmission cycles include species such as western gray squirrels and dusky-footed woodrats [78]; transmission to these hosts was not measured in the present study but may be higher given the higher infection prevalence measured for these species relative to *P. maniculatus* [42]. Further, as we could not test the infection status of each tick prior to loading on a host, the number of infected ticks applied to each C3H/HeN mouse, when measured after feeding, were incidentally higher for *I. scapularis* than *I. pacificus* and varied between mouse replicates (Table 2). Thus, variation in infected tick abundance and lack of shared history between host and vector species may confound the results obtained here or limit their ecological relevance. However, the C3H/HeN mouse model is routinely used in *B. burgdorferi* transmission studies as this species is highly capable of acquiring infection [79–82] and does not develop resistance to *I. pacificus* infestation in laboratory trials [83]. Further, heavier tick infestations can generate stronger host immune responses [84], thus the effect of loading more *I. scapularis* on mice relative to *I. pacificus*, does not necessarily favor higher transmission from *I. scapularis*. Overall, while the experimental conditions do not exactly mirror those of natural environments, the highly contrasting transmission efficiencies of *I. scapularis* and *I. pacificus* observed here highlight vector-to-host transmission as a vector trait potentially driving differences in transmission efficiency between the eastern and western USA.

These same differences between *I. scapularis* and *I. pacificus* were not observed when comparing pathogen acquisition abilities. An identical proportion of *I. scapularis* and *I. pacificus* acquired their sympatric pathogen strain (B31 and CA4, respectively) when fed on infected mice. Pathogen acquisition efficiency was also identical between *I. scapularis* and *I. pacificus* for their respective allopatric strain, although lower than that of their sympatric strain. These results indicate that *I. scapularis* and *I. pacificus* are equally capable of acquiring pathogens from infected hosts, and highlight the importance of coevolutionary history between vectors and pathogen strain in vector competence.

Given their overlapping life history strategies, vectors and pathogens form intimate relationships [85]. These tight relationships can evolve into positive [86–92] or negative [93–98] associations, with outcomes often varying based on the degree and duration of evolutionary association [89]. In laboratory experiments, *B. burgdorferi* infection has been shown to promote nymphal survival and host-seeking under suboptimal environmental conditions [99, 100], suggesting a co-evolved mutualistic relationship. Our finding that sympatric vector species and *B. burgdorferi* strains exhibit more efficient pathogen dynamics than allopatric pairings further supports this notion. However, recent genomic work found no support for coevolution between *B. burgdorferi* and its tick vector [101]. The results found in this study may thus reflect nascent mutualistic associations arising between *B. burgdorferi* and ixodid vectors not yet reflected in their phylogenetic structure. However, we did not directly measure the effect of pathogen infection on tick behavior, survivorship, or reproductive fitness. Given the species-specific differences we document in this study, future studies should evaluate the pathogenic impact of *B. burgdorferi* infection on tick fitness. Further investigation of coevolution between ixodid vectors and *B. burgdorferi* strains are needed to better understand the role of coevolution in current transmission dynamics.

While our common-garden laboratory experiments revealed significant differences in pathogen dynamics and host attachment rates between *I. scapularis* and *I. pacificus*, these results may not reflect natural differences in these tick species in all regions and populations. In particular, as our *I. scapularis* and *I. pacificus* populations were sourced from Rhode Island and California, respectively, these results may not extend to southern populations of either species, as these are known to be genetically and ecologically distinct [102, 103]. However, northeastern and midwestern *I. scapularis* populations lack significant genetic structuring, as do northern *I. pacificus* populations [102, 103], suggesting the tick species-specific differences detected in our study are likely applicable to tick populations in areas with higher Lyme disease incidence. Further, Lyme disease transmission to humans is rare in the southeastern and southwestern USA, as vector populations here are less likely to quest above the leaf litter due to warmer and drier climate conditions and genetic differences in these populations [20, 104–109]. Thus, our results indicating vector-specific differences also contribute to variation in Lyme disease transmission efficiency may be most relevant in regions that are otherwise climatically and ecologically suitable for disease transmission.

## Conclusions

Despite high genetic similarity between the two USA Lyme disease vectors, we found significant variation in *I. scapularis* and *I. pacificus* performance in traits related to vector competence. *Ixodes pacificus* displayed greater host feeding abilities, but far lower pathogen transmission rates relative to *I. scapularis*. Inefficient tick to host transmission may present a barrier to *B. burgdorferi* transmission cycles in the western USA, contributing to broad regional differences in Lyme disease incidence. Host to tick transmission rates were identical between tick species, but lower for allopatric tick species and pathogen strains than sympatric pairings. These findings highlight the importance of vector-pathogen coevolutionary history, as well as vector traits, in determining Lyme disease transmission efficiency.

## Supplementary information

**Additional file 1: Table S1.** Host attachment rates for larval *I. pacificus* and *I. scapularis* fed on naïve *Peromyscus maniculatus.* 150 larvae (either *I. pacificus* or *I. scapularis*) were placed on each of four mice.

**Additional file 2: Table S2.** Weights of unengorged and engorged larval *I. pacificus* and *I. scapularis* (mg).

## Data Availability

All data generated or analyzed during this study are included in this published article and its additional files.

## Abbreviations

PCR: polymerase chain reaction
DNA: deoxyribonucleic acid
BSK-II: Barbour–Stoenner–Kelley
