# Supplementary material for: Comparative vector competence of North American Lyme disease vectors
Source: Parasit Vectors. 2020 Jan 14;13:29. doi: 10.1186/s13071-020-3893-x (PMC6961398; doi:10.1186/s13071-020-3893-x)
Supplement: Supplementary file 1 — Additional file 1: Table S1. Host attachment rates for larval I. pacificus and I. scapularis fed on naïve Peromyscus maniculatus. 150 larvae (either I. pacificus or I. scapularis) were placed on each of four mice. [file 13071_2020_3893_MOESM1_ESM.docx]

**Additional file 1: Table S1.** Host attachment rates for larval *I. pacificus* and *I. scapularis* fed on naïve *Peromyscus maniculatus.* 150 larvae (either *I. pacificus* or *I. scapularis*) were placed on each of four mice.

|  | **Mouse A** | **Mouse B** | **Mouse C** | **Mouse D** |
| --- | --- | --- | --- | --- |
| **Tick Species** | ***I. pacificus*** | ***I. pacificus*** | ***I. scapularis*** | ***I. scapularis*** |
| Day 1 count | 0 | 0 | 0 | 0 |
| Day 2 count | 0 | 0 | 0 | 0 |
| Day 3 count | 25 | 24 | 11 | 6 |
| Day 4 count | 17 | 18 | 1 | 2 |
| Day 5 count | 7 | 8 | 0 | 0 |
| Day 6 count | 1 | 2 | 0 | 0 |
