# Supplementary material for: Comparative vector competence of North American Lyme disease vectors
Source: Parasit Vectors. 2020 Jan 14;13:29. doi: 10.1186/s13071-020-3893-x (PMC6961398; doi:10.1186/s13071-020-3893-x)
Supplement: Supplementary file 2 — Additional file 2: Table S2. Weights of unengorged and engorged larval I. pacificus and I. scapularis (mg). [file 13071_2020_3893_MOESM2_ESM.docx]

**Additional file 2: Table S2.** Weights of unengorged and engorged larval *I. pacificus* and *I. scapularis* (mg).

| **Species** | **Engorgement Status** | **Mouse Replicate** | **Weight (mg)** |
| --- | --- | --- | --- |
| *I.pacificus* | Engorged | A | 0.53 |
| *I.pacificus* | Engorged | A | 0.5 |
| *I.pacificus* | Engorged | A | 0.38 |
| *I.pacificus* | Engorged | A | 0.41 |
| *I.pacificus* | Engorged | A | 0.32 |
| *I.pacificus* | Engorged | A | 0.43 |
| *I.pacificus* | Engorged | A | 0.41 |
| *I.pacificus* | Engorged | A | 0.63 |
| *I.pacificus* | Engorged | A | 0.61 |
| *I.pacificus* | Engorged | A | 0.67 |
| *I.pacificus* | Engorged | A | 0.76 |
| *I.pacificus* | Engorged | A | 0.66 |
| *I.pacificus* | Engorged | A | 0.77 |
| *I.pacificus* | Engorged | A | 0.7 |
| *I.pacificus* | Engorged | A | 0.86 |
| *I.pacificus* | Engorged | A | 0.58 |
| *I.pacificus* | Engorged | A | 0.55 |
| *I.pacificus* | Engorged | A | 0.49 |
| *I.pacificus* | Engorged | A | 0.56 |
| *I.pacificus* | Engorged | A | 0.53 |
| *I.pacificus* | Engorged | A | 0.52 |
| *I.pacificus* | Engorged | A | 0.47 |
| *I.pacificus* | Engorged | A | 0.2 |
| *I.pacificus* | Engorged | B | 0.36 |
| *I.pacificus* | Engorged | B | 0.42 |
| *I.pacificus* | Engorged | B | 0.71 |
| *I.pacificus* | Engorged | B | 0.42 |
| *I.pacificus* | Engorged | B | 0.42 |
| *I.pacificus* | Engorged | B | 0.31 |
| *I.pacificus* | Engorged | B | 0.37 |
| *I.pacificus* | Engorged | B | 0.33 |
| *I.pacificus* | Engorged | B | 0.35 |
| *I.pacificus* | Engorged | B | 0.39 |
| *I.pacificus* | Engorged | B | 0.38 |
| *I.pacificus* | Engorged | B | 0.32 |
| *I.pacificus* | Engorged | B | 0.44 |
| *I.pacificus* | Engorged | B | 0.38 |
| *I.pacificus* | Engorged | B | 0.34 |
| *I.scapularis* | Engorged | C | 0.36 |
| *I.scapularis* | Engorged | C | 0.35 |
| *I.scapularis* | Engorged | C | 0.27 |
| *I.scapularis* | Engorged | C | 0.22 |
| *I.scapularis* | Engorged | C | 0.16 |
| *I.scapularis* | Engorged | C | 0.25 |
| *I.scapularis* | Engorged | C | 0.13 |
| *I.scapularis* | Engorged | C | 0.27 |
| *I.scapularis* | Engorged | C | 0.25 |
| *I.scapularis* | Engorged | C | 0.15 |
| *I.scapularis* | Engorged | D | 0.39 |
| *I.scapularis* | Engorged | D | 0.4 |
| *I.scapularis* | Engorged | D | 0.32 |
| *I.scapularis* | Engorged | D | 0.24 |
| *I.pacificus* | Engorged | A | 0.4 |
| *I.pacificus* | Engorged | A | 0.37 |
| *I.pacificus* | Engorged | A | 0.28 |
| *I.pacificus* | Engorged | A | 0.33 |
| *I.pacificus* | Engorged | A | 0.27 |
| *I.pacificus* | Engorged | A | 0.3 |
| *I.pacificus* | Engorged | A | 0.36 |
| *I.pacificus* | Engorged | A | 0.32 |
| *I.pacificus* | Engorged | A | 0.19 |
| *I.pacificus* | Engorged | A | 0.3 |
| *I.pacificus* | Engorged | A | 0.38 |
| *I.pacificus* | Engorged | A | 0.4 |
| *I.pacificus* | Engorged | A | 0.37 |
| *I.pacificus* | Engorged | A | 0.35 |
| *I.pacificus* | Engorged | A | 0.42 |
| *I.pacificus* | Engorged | A | 0.28 |
| *I.pacificus* | Engorged | B | 0.38 |
| *I.pacificus* | Engorged | B | 0.33 |
| *I.pacificus* | Engorged | B | 0.29 |
| *I.pacificus* | Engorged | B | 0.36 |
| *I.pacificus* | Engorged | B | 0.38 |
| *I.pacificus* | Engorged | B | 0.32 |
| *I.pacificus* | Engorged | B | 0.37 |
| *I.pacificus* | Engorged | B | 0.44 |
| *I.pacificus* | Engorged | B | 0.41 |
| *I.pacificus* | Engorged | B | 0.31 |
| *I.pacificus* | Engorged | B | 0.39 |
| *I.pacificus* | Engorged | B | 0.41 |
| *I.pacificus* | Engorged | B | 0.42 |
| *I.pacificus* | Engorged | B | 0.32 |
| *I.pacificus* | Engorged | B | 0.35 |
| *I.scapularis* | Engorged | C | NA |
| *I.scapularis* | Engorged | D | 0.21 |
| *I.pacificus* | Engorged | A | 0.36 |
| *I.pacificus* | Engorged | A | 0.34 |
| *I.pacificus* | Engorged | B | 0.33 |
| *I.pacificus* | Engorged | B | 0.39 |
| *I.pacificus* | Engorged | B | 0.21 |
| *I.pacificus* | Engorged | A | 0.36 |
| *I.pacificus* | Engorged | B | 0.25 |
| *I.pacificus* | Engorged | B | 0.2 |
| *I.pacificus* | Unengorged | NA | 0.02 |
| *I.pacificus* | Unengorged | NA | 0.022 |
| *I.pacificus* | Unengorged | NA | 0.024 |
| *I.pacificus* | Unengorged | NA | 0.022 |
| *I.pacificus* | Unengorged | NA | 0.024 |
| *I.pacificus* | Unengorged | NA | 0.026 |
| *I.pacificus* | Unengorged | NA | 0.016 |
| *I.pacificus* | Unengorged | NA | 0.024 |
| *I.pacificus* | Unengorged | NA | 0.018 |
| *I.pacificus* | Unengorged | NA | 0.032 |
| *I.pacificus* | Unengorged | NA | 0.018 |
| *I.pacificus* | Unengorged | NA | 0.018 |
| *I.pacificus* | Unengorged | NA | 0.018 |
| *I.pacificus* | Unengorged | NA | 0.026 |
| *I.pacificus* | Unengorged | NA | 0.022 |
| *I.pacificus* | Unengorged | NA | 0.026 |
| *I.pacificus* | Unengorged | NA | 0.036 |
| *I.pacificus* | Unengorged | NA | 0.036 |
| *I.pacificus* | Unengorged | NA | 0.02 |
| *I.pacificus* | Unengorged | NA | 0.02 |
| *I.pacificus* | Unengorged | NA | 0.024 |
| *I.pacificus* | Unengorged | NA | 0.02 |
| *I.pacificus* | Unengorged | NA | 0.018 |
| *I.pacificus* | Unengorged | NA | 0.018 |
| *I.pacificus* | Unengorged | NA | 0.018 |
| *I.pacificus* | Unengorged | NA | 0.022 |
| *I.pacificus* | Unengorged | NA | 0.022 |
| *I.pacificus* | Unengorged | NA | 0.24 |
| *I.pacificus* | Unengorged | NA | 0.02 |
| *I.pacificus* | Unengorged | NA | 0.022 |
| *I.pacificus* | Unengorged | NA | 0.018 |
| *I.pacificus* | Unengorged | NA | 0.026 |
| *I.pacificus* | Unengorged | NA | 0.022 |
| *I.pacificus* | Unengorged | NA | 0.022 |
| *I.pacificus* | Unengorged | NA | 0.028 |
| *I.pacificus* | Unengorged | NA | 0.024 |
| *I.pacificus* | Unengorged | NA | 0.024 |
| *I.pacificus* | Unengorged | NA | 0.02 |
| *I.pacificus* | Unengorged | NA | 0.02 |
| *I.pacificus* | Unengorged | NA | 0.022 |
| *I.scapularis* | Unengorged | NA | 0.05 |
| *I.scapularis* | Unengorged | NA | 0.044 |
| *I.scapularis* | Unengorged | NA | 0.052 |
| *I.scapularis* | Unengorged | NA | 0.048 |
| *I.scapularis* | Unengorged | NA | 0.05 |
| *I.scapularis* | Unengorged | NA | 0.054 |
| *I.scapularis* | Unengorged | NA | 0.05 |
| *I.scapularis* | Unengorged | NA | 0.056 |
| *I.scapularis* | Unengorged | NA | 0.042 |
| *I.scapularis* | Unengorged | NA | 0.048 |
| *I.scapularis* | Unengorged | NA | 0.046 |
| *I.scapularis* | Unengorged | NA | 0.058 |
| *I.scapularis* | Unengorged | NA | 0.022 |
| *I.scapularis* | Unengorged | NA | 0.028 |
| *I.scapularis* | Unengorged | NA | 0.026 |
| *I.scapularis* | Unengorged | NA | 0.026 |
| *I.scapularis* | Unengorged | NA | 0.028 |
| *I.scapularis* | Unengorged | NA | 0.016 |
| *I.scapularis* | Unengorged | NA | 0.01 |
| *I.scapularis* | Unengorged | NA | 0.03 |
| *I.scapularis* | Unengorged | NA | 0.022 |
| *I.scapularis* | Unengorged | NA | 0.03 |
| *I.scapularis* | Unengorged | NA | 0.012 |
| *I.scapularis* | Unengorged | NA | 0.04 |
| *I.scapularis* | Unengorged | NA | 0.026 |
| *I.scapularis* | Unengorged | NA | 0.028 |
| *I.scapularis* | Unengorged | NA | 0.028 |
| *I.scapularis* | Unengorged | NA | 0.026 |
| *I.scapularis* | Unengorged | NA | 0.026 |
| *I.scapularis* | Unengorged | NA | 0.022 |
| *I.scapularis* | Unengorged | NA | 0.036 |
| *I.scapularis* | Unengorged | NA | 0.044 |
| *I.scapularis* | Unengorged | NA | 0.018 |
| *I.scapularis* | Unengorged | NA | 0.024 |
| *I.scapularis* | Unengorged | NA | 0.026 |
| *I.scapularis* | Unengorged | NA | 0.028 |
| *I.scapularis* | Unengorged | NA | 0.024 |
| *I.scapularis* | Unengorged | NA | 0.018 |
| *I.scapularis* | Unengorged | NA | 0.036 |
| *I.scapularis* | Unengorged | NA | 0.04 |
| *I.scapularis* | Unengorged | NA | 0.018 |
| *I.scapularis* | Unengorged | NA | 0.036 |
| *I.scapularis* | Unengorged | NA | 0.026 |
| *I.scapularis* | Unengorged | NA | 0.036 |
| *I.scapularis* | Unengorged | NA | 0.042 |
| *I.scapularis* | Unengorged | NA | 0.03 |
